# Supplementary material for: A small-molecule P2RX7 activator promotes anti-tumor immune responses and sensitizes lung tumor to immunotherapy
Source: Nat Commun. 2021 Jan 28;12:653. doi: 10.1038/s41467-021-20912-2 (PMC7843983; doi:10.1038/s41467-021-20912-2)
Supplement: Supplementary file 1 — Supplementary Information [file 41467_2021_20912_MOESM1_ESM.pdf]

Supplementary Table 1: Antibodies used in this study

Antibody	Compagny	Clone	*Species*	Isotype	Fluorochrome	Stock Conc.	Dilution
CD16/CD32 Fc Block	BD Biosciences	2.4G2	*Rat SD (outbread)*	IgG2B. κ	Uncoupled	0.5mg/ml	1/100
CD3ε	BD Biosciences	145-2611	*Armenian Hamster*	IgG1. κ	PerCP-Cy5.5	0.2mg/ml	1/100
CD4	Biolegend	GK1.5	*Rat*	IgG2b. κ	AF647	0.5 mg/ml	1/100
CD4	BD Biosciences	GK1.5	*Rat LEW*	IgG2b. κ	PE	0.2mg/ml	1/100
CD4	BD Biosciences	RM4-5	*Rat DA*	IgG2a. κ	BV711	0.2mg/ml	1/100
CD8α	Biolegend	53-6.7	*Rat*	IgG2a. κ	BV650	50µg/ml	1/100
CD8α	BD Biosciences	53-6.7	*Rat LOU*	IgG2a. κ	BV650	0.2mg/ml	1/100
γδ TCR	BD Biosciences	GL3	*Armenian Hamster*	IgG2. κ	PE	0.2mg/ml	1/100
CD25	BD Biosciences	PC61	*Rat OFA*	IgG1. λ	V450	0.2mg/ml	1/100
CD44	BD Biosciences	IM7	*Rat*	IgG2. κ	APC	0.2mg/ml	1/100
CD44	BD Biosciences	IM7	*Rat*	IgG2. κ	PE-Cy7	0.2mg/ml	1/100
CCR7	BD Biosciences	4B12	*Rat LOU*	IgG2a.	P2-CF594	0.2mg/ml	1/100
CD107a	BD Biosciences	1D4B	*Rat SD (outbred)*	IgG2a. κ	PE-Cy7	0.2mg/ml	1/100
NK1.1	BD Biosciences	PK136	*Mouse C3H x BALB/c*	IgG2a. κ	PE-CF594	0.2mg/ml	1/100
B220	eBiosciences	RA3-6B2	*Rat*	IgG2a. κ	FITC	0.5 mg/ml	1/200
CD19	BD Biosciences	ID3	*Rat LEW*	IgG2a. κ	FITC	0.5 mg/ml	1/100
CD45.2	BD Biosciences	104	*Mouse SJL*	IgG2a. κ	BV786	0.2mg/ml	1/100
CD11b	eBiosciences	M1/70	*Rat*	IgG2b. κ	APC	0.2mg/ml	1/400
CD11c	BD Biosciences	HL3	*Armenian Hamster*	IgG1. λ2	PE-Cy7	0.2mg/ml	1/100
Ly6C	BD Biosciences	AL-21	*Rat*	IgM. κ	V450	0.2mg/ml	1/100
Ly6G	BD Biosciences	1A8	*Rat LEW*	IgG2a. κ	AF700	0.2mg/ml	1/100
Ly6G	BD Biosciences	1A8	*Rat LEW*	IgG2a. κ	FITC	0.5mg/ml	1/100
CD80	Biolegend	16-10A1	*Armenian Hamster*	IgG	PE	0.2mg/ml	1/100
CD86	BD Biosciences	GL1	*Rat LOU*	IgG2a. κ	AF700	0.2mg/ml	1/100
CD86	BD Biosciences	GL1	*Rat LOU*	IgG2a. κ	APC	0.2mg/ml	1/100
I-A/I-E	Biolegend	M5/114.15.2	*Rat*	IgG2b. κ	APCfire750	0.2mg/ml	1/100
H-2Kb/H-2Db	Biolegend	28//8//6	*Mouse (C3H)*	IgG2a. κ	FITC	0.5mg/ml	1/100
IL-10	BD Biosciences	JES5-16E3	*Rat*	IgG2b	BV421	0.2mg/ml	1/100
Foxp3	eBiosciences	FJK16S	*Rat*	IgG2a. κ	PE	0.2mg/ml	1/100
CD103	Biolegend	2E/7	*Armenian Hamster*	IgG	PerCP-Cy5.5	0.2mg/ml	1/100
IFNγ	BD Biosciences	XMG1.2	*Rat*	IgG1. κ	APC	0.2mg/ml	1/100
IL-17A	BD Biosciences	TC11-18H10	*Armenian Hamster*	IgG1. κ	AF700	0.2mg/ml	1/100
IL-4	BD Biosciences	11B11	*Rat*	IgG1	BV711	0.2mg/ml	1/100
IL-13	eBiosciences	eBio13A	*Rat*	IgG1. κ	AF488	0.5 mg/ml	1/100
GATA3	BD Biosciences	L50-823	*Mouse BALB/c*	IgG1. κ	BV421	0.2mg/ml	1/100
CD279 (PD1)	BD Biosciences	J43	*Rat*	IgG2a. κ	APC	0.2mg/ml	1/100
CD274 (PD-L1)	Biolegend	10F.9G2	*Rat*	IgG2b. κ	APC	0.2mg/ml	1/100
CD274 (PD-L1)	Biolegend	10F.9G2	*Rat*	IgG2b. κ	BV421	100µg/ml	1/100
CD273 (PD-L2)	Biolegend	TY25	*Rat*	IgG2a. κ	PEdazzle594	0.2mg/ml	1/100
CTLA4	BD Biosciences	UC10-4F10-11	*Armenian Hamster*	IgG1. κ	PE	0.2mg/ml	1/100
TIM-3	BD Biosciences	5D12/TIM-3	*Mouse*	IgG1. κ	PE	0.2mg/ml	1/100
P2RX7	Biolegend	1F11	*Rat*	IgG2b. κ	PE	0.2mg/ml	1/8
IL-1β	BioXCell	B122	*Armenian Hamster*	IgG	na	na	200µg
IL-18	BioXCell	YIGIF74-1G7	*Rat*	IgG2b. κ	na	na	200µg
CD-8	BioXCell	53-6.7	*Rat*	IgG2a. κ	na	na	200µg
CD-4	BioXCell	GK1.5	*Rat*	IgG2b. κ	na	na	200µg
NK1.1	BioXCell	PK136	*Mouse*	IgG2a. κ	na	na	300µg
αPD-1	BioXCell	RPM1-4	*Rat*	IgG2a. κ	na	na	200µg
CD3	Roche	2GV6	*Rabbit*	IgG	na	Ready to use	na
CD8	Roche	SP57	*Rabbit*	IgG	na	Ready to use	na
IL-18	BioVision	na	*Rabbit*	IgG	na	0.5mg/ml	1/200
αPD-1	Dako	22C3	*Mouse*	IgG1	na	150mg/L	1/50
Ki67	Abcam	SP6	*Rabbit*	IgG	na	1mg/ml	1/100
NLRP3	Adipogen	Cryo-2	*Mouse*	IgG2b	na	1mg/ml	1/1000
ASC	Adipogen	AL177	*Rabbit*	IgG	na	1mg/ml	1/1000
Caspase 1	Adipogen	Casper-1	*Mouse*	IgG1	na	1mg/ml	1/1000
ACTB	Biorad	VMA00048	*Mouse*	IgG1	na	1mg/ml	1/60000
P2RX7	Alomone Labs	APR-008	*Rabbit*	IgG	na	1mg/ml	1/1000

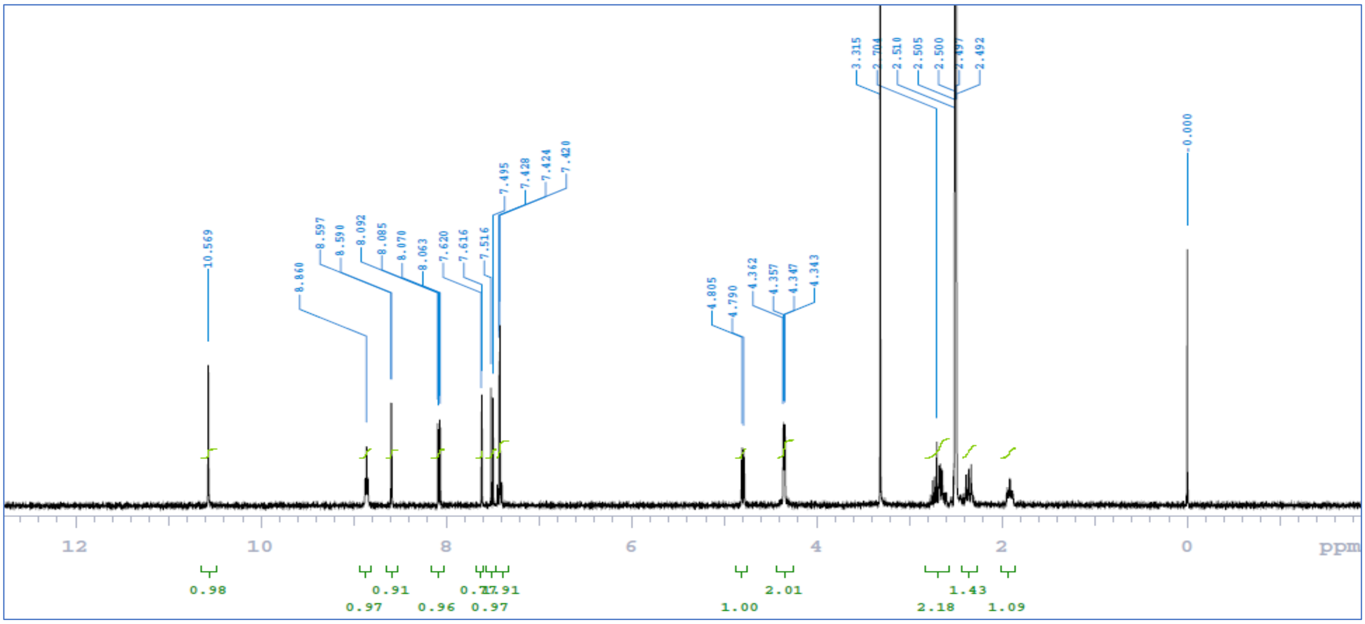

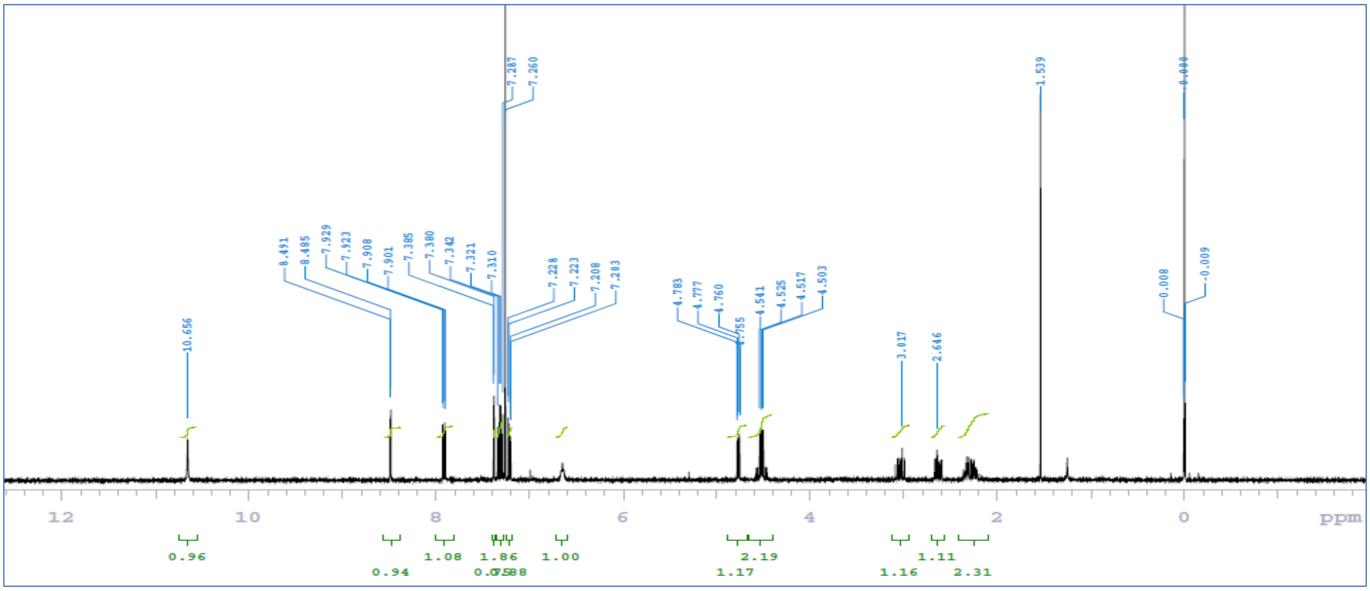

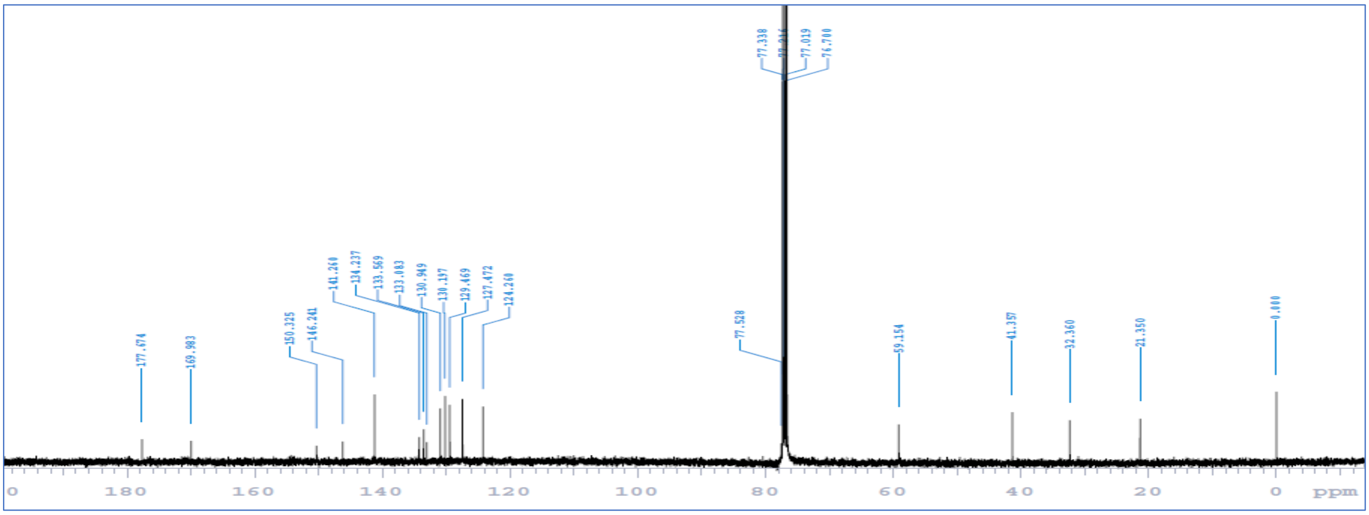

Supplementary Fig. 1. Spectra ^{1}H and ^{13}C NMR of synthesized acylurea HEI3090. Representative image of Spectra ^{1}H in DMSO-d_6 and $CDCl_3$ and ^{13}C in $CDCl_3$ of (S)-N^{1}-(6-chloropyridin-3-yl)-N^{2}-(2,4-dichlorobenzyl)-5-oxopyrrolidine-1,2-dicarboxamide (HEI3090)

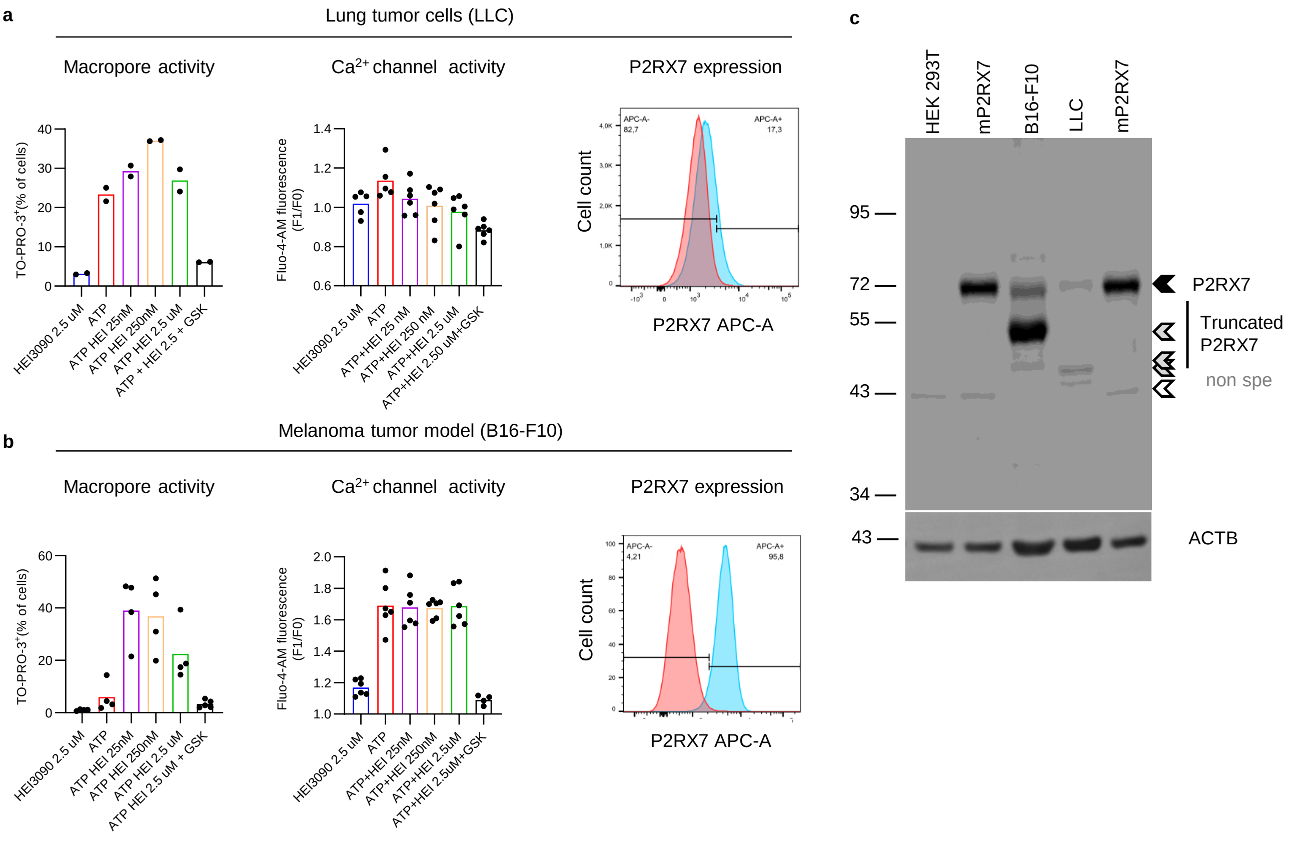

Supplementary Fig. 2. P2RX7 is expressed on LLC and B16-F10 tumor cells

P2RX7 characterization in Lewis Lung carcinoma (LLC) (**a**) and melanoma (B16-F10) cell lines (**b**) showing that tumor cell line express a functional P2RX7 (macropore and channel activity). Macropore activity: Tumor cells were loaded with TO-PRO-3 and stimulated with 500 µM ATP alone or in the presence of HEI3090 or GSK 1370319A, a P2RX7 antagonist. At 15min post stimulation an image was taken and TO-PRO-3+ cells were counted. Data are presented as scatter dot plots (n = >1000 cells examined over 2 independent experiments). The P2RX7's Ca^{2+} channel activity we measured the ATP-induced increase of intracellular Ca^{2+} concentration (Fluo-4-AM uptake) in the presence of the P2RX7's inhibitor (GSK1370319A), using a plate reader. Data are presented as scatter dot plots (n=2 independent experiments, in triplicate). P2RX7 expression was assayed by flow cytometry (n= 100000 cells examined over 2 independent experiments) using the anti P2RX7 antibody (clone 1F11, right panel **a** and **b**) or by western blotting using an anti-extracellular loop of P2RX7 antibody (APR-008, Alomone Labs) that recognizes a band of 70kDa, the expected size for P2RX7 (1 experiment performed) (**c**). These results demonstrated that both cell lines expressed an active P2RX7, with B16-F10 cells expressing higher P2RX7 levels than LLC cells. Further we showed that tumor cell lines express truncated P2RX7's isoforms and that HEI3090 only impacts the macropore activity on these P2RX7 isoforms. Source data are provided as a Source Data file.

Vehicle

HEI3090

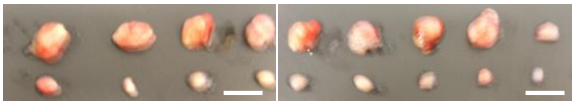

Supplementary Fig. 3: HEI3090 effect on LLC tumor size
$5x10^5$ LLC were injected s.c. into the flank of C57BL6/J mice. Mice were injected i.p every day with vehicle or HEI3090 (1.5 mg/kg) from day 1 to day 12. Representative picture of tumors from Fig 2a showing tumor size the day of the sacrifice. Bar = 10 mm

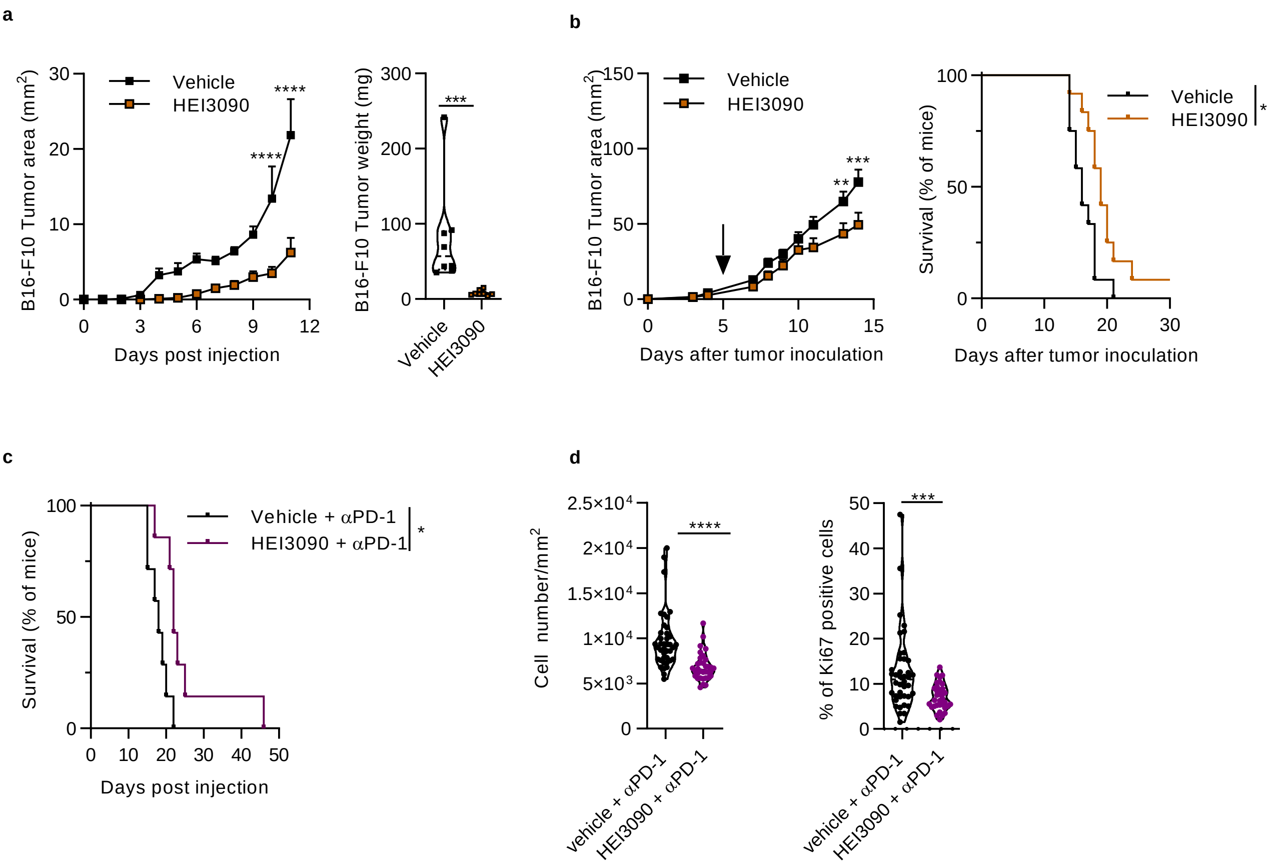

Supplementary Fig. 4: HEI3090 inhibits the growth of melanoma B16-F10 tumor cells and increased mice survival when combined with anti-PD-1 antibody

a. 5x10^5 B16-F10 were injected s.c. into the flank of C57BL6/J mice. Mice were injected i.p every day with vehicle or HEI3090 (1.5 mg/kg) from day 1 to day 12. Curves showed mean tumor area in mm^2 ± SEM (n= 8 mice, Two-way Anova test, left panel) and graph showed tumor weight the day of sacrifice. Data are presented by violin plots showing all points with hatched bar corresponding to median tumor weight (n= 8 mice, Two-tailed Mann Whitney test, right panel). **b.** Effect of HEI3090 in therapeutic administration. Mice were treated i.p. with vehicle or HEI3090 (3 mg/kg) from days 5 (when tumors reached a volume of approximatively 5-10 mm^2) to 15. Curves showed mean tumor area in mm^2 ± SEM (n= 12 mice, Two-way Anova test, left panel) and survival of B16-F10 tumor bearing mice (n= 8 mice, Mantel Cox test, right panel). **c.** Effect of HEI3090 and αPD-1 checkpoint inhibitor on survival of B16-F10 tumor-bearing mice. 5x10^5 B16-F10 were injected s.c. into the flank of WT mice. Mice were treated i.p. with vehicle or with HEI3090 (3 mg/kg) from day 1 to day 18. Each mouse received 200 µg of αPD-1 i.p. at day 4, 7, 10, 12 and 16. Mice were sacrificed when tumor reached 100 mm^2. (n= 7 mice, Mantel Cox test). **d**. Efficacy of the combo treatment in the *in situ* (LSL *KRas^{G12D}*) genetic lung tumor mouse model. Ten lung lesions per mouse (5 large, 5 small) were selected. Average cell number/mm^2 and Ki67$^+$ cells are shown. Data are presented by violin plots showing all individual lesion to overcome the individual heterogeneity of each lesion, with plain bar corresponding to the median (n= 5 mice, Two-tailed Mann Whitney test). p-values: *p<0.05, **p<0.01 ***p<0.001, ****p<0.0001. Source data are provided as a Source Data file.

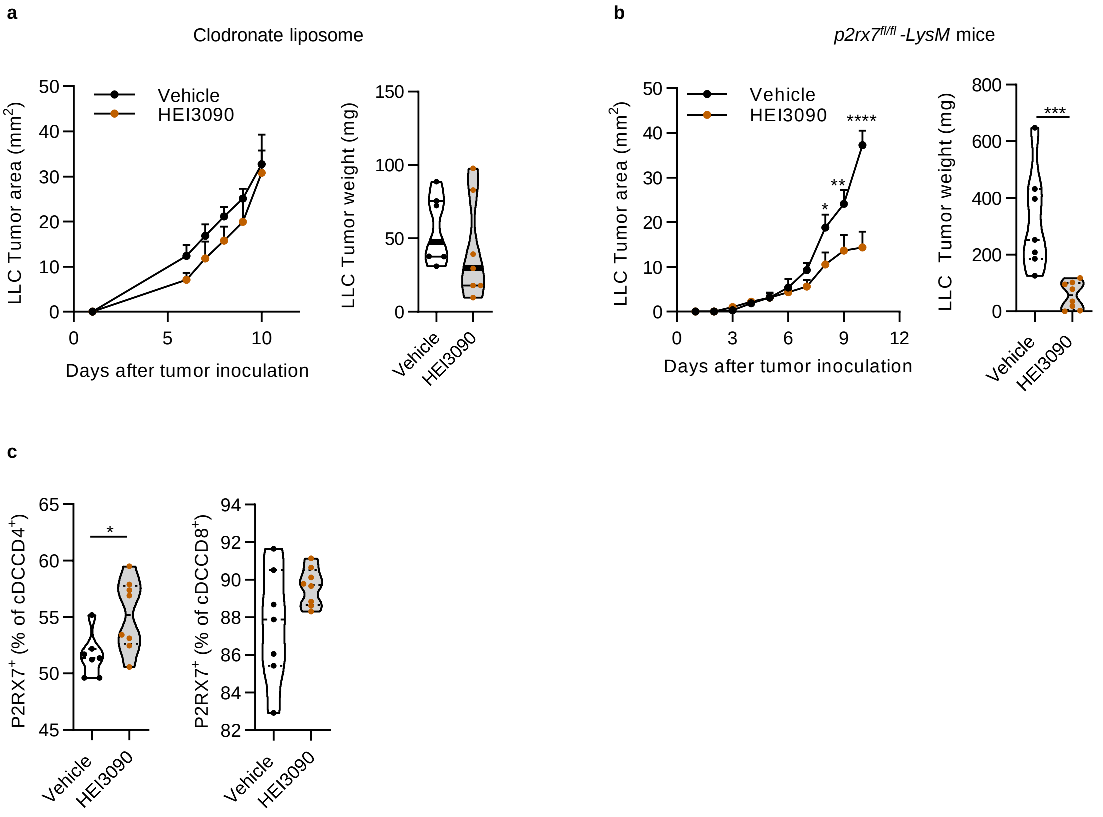

Supplementary Fig. 5: HEI3090 targets P2RX7-expressing dendritic cells.
a. Cells from the myeloid lineage do not support HEI3090-induced antitumor response. 5x10^5 LLC cells were injected s.c. to *p2rx7*$^{fl/fl}$-LysM mice and mice were daily treated with HEI3090 or vehicle, as detailed in the method section. Curves showed mean tumor area in mm^2 ± SEM (n= 7 mice, Two-way Anova test, left panel) and graph showed tumor weight the day of sacrifice. Data are presented by violin plots showing all points with hatched bar corresponding to the median of tumor weight (n= 7 mice, Two-tailed Mann Whitney test, right panel). **b.** Three days prior to tumor cell inoculation, liposome clodronate (200 µl) was injected i.p. to mice. Mice were then injected every 3 days with liposome clodronate 1h before HEI3090 treatment. 5.10^5 LLC cells were inoculated s.c. and mice were treated daily with 1.5 mg/kg of HEI3090 or vehicle. Tumor area was measured with a caliper. At the end of the experiment, tumors were weighted. Curves showed mean tumor area in mm^2 ± SEM (n= 7 mice, Two-way Anova test, left panel) and graph showed tumor weight the day of sacrifice. Data are presented by violin plots showing all points with hatched bar corresponding to the median of tumor weight (n= 7 mice, Two-tailed Mann Whitney test, right panel). **c.** P2RX7 expression by DC $CD4^+$ cells is increased in response to HEI3090. Data are presented by violin plots showing the percentage of $P2RX7^+$ cells of indicated cells, with hatched bar corresponding to the median of positive cells (n= 6 mice, Two-tailed Mann Whitney test). p-values: *p<0.05, **p<0.01 ***p<0.001, ****p<0.0001. Source data are provided as a Source Data file.

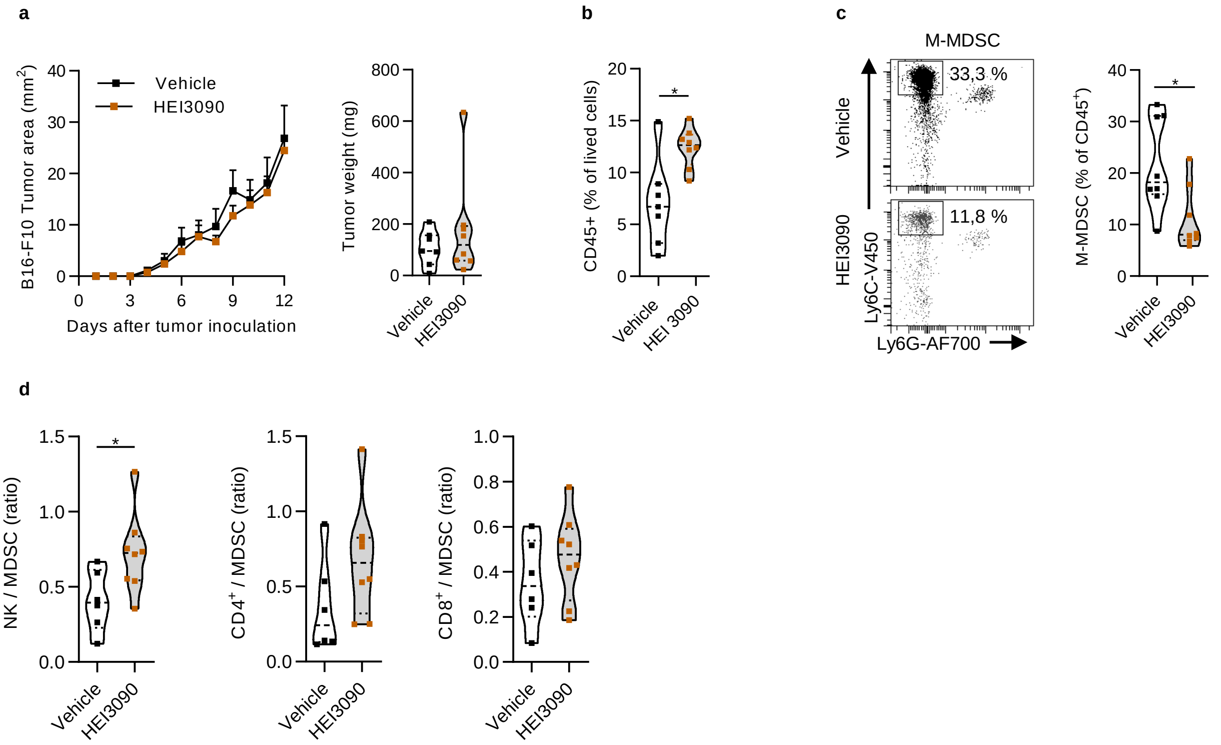

Supplementary Fig. 6: Immune cells mediate the antitumor activity induced by HEI3090 in the B16-F10 tumor mouse model
a. Effect of HEI3090 on B16-F10 tumor growth in *p2rx7*$^{-/-}$ mice. 5x10^5 cells were injected s.c. into the flank of *p2rx7*$^{-/-}$ mice. Mice were treated i.p. with vehicle or with HEI3090 (1.5 mg/kg). Curves showed mean tumor area in mm^2 ± SEM (n= 7 mice, Two-way Anova test, left panel) and graph showed tumor weight the day of sacrifice. Data are presented by violin plots showing all points with hatched bar corresponding to the median of tumor weight (n= 7 mice, Two-tailed Mann Whitney, right panel). **b.** Effect of HEI3090 on the recruitment of immune cells within the TME. 5x10^5 B16-F10 cells were injected s.c. into the flank of WT mice. Mice were injected i.p every day with vehicle or HEI3090. At day 12, tumors were collected for flow cytometry analyses. Data are presented by violin plots showing all points with hatched bar corresponding to the median of CD45$^+$ cells of total lived cells (n= 7 mice, Two-tailed Mann Whitney test). **c.** Proportion of M-MDSC within the TME among CD45$^+$ within B16-F10 tumors from mice treated with vehicle or HEI3090. Gating strategy is shown in left panel. Data are presented by violin plots showing all points with hatched bar corresponding to the median of M-MDSC cells over CD45$^+$ cells (n= 7 mice, Two-tailed Mann Whitney test). **d.** Ratio of NK, CD4$^+$ or CD8$^+$ T cells over M-MDSC within the TME. Data are presented by violin plots showing all points with hatched bar corresponding to the median of indicated cells of MDSC cells (n= 7 mice, Two-tailed Mann Whitney).). p-values: *p<0.05. Source data are provided as a Source Data file.

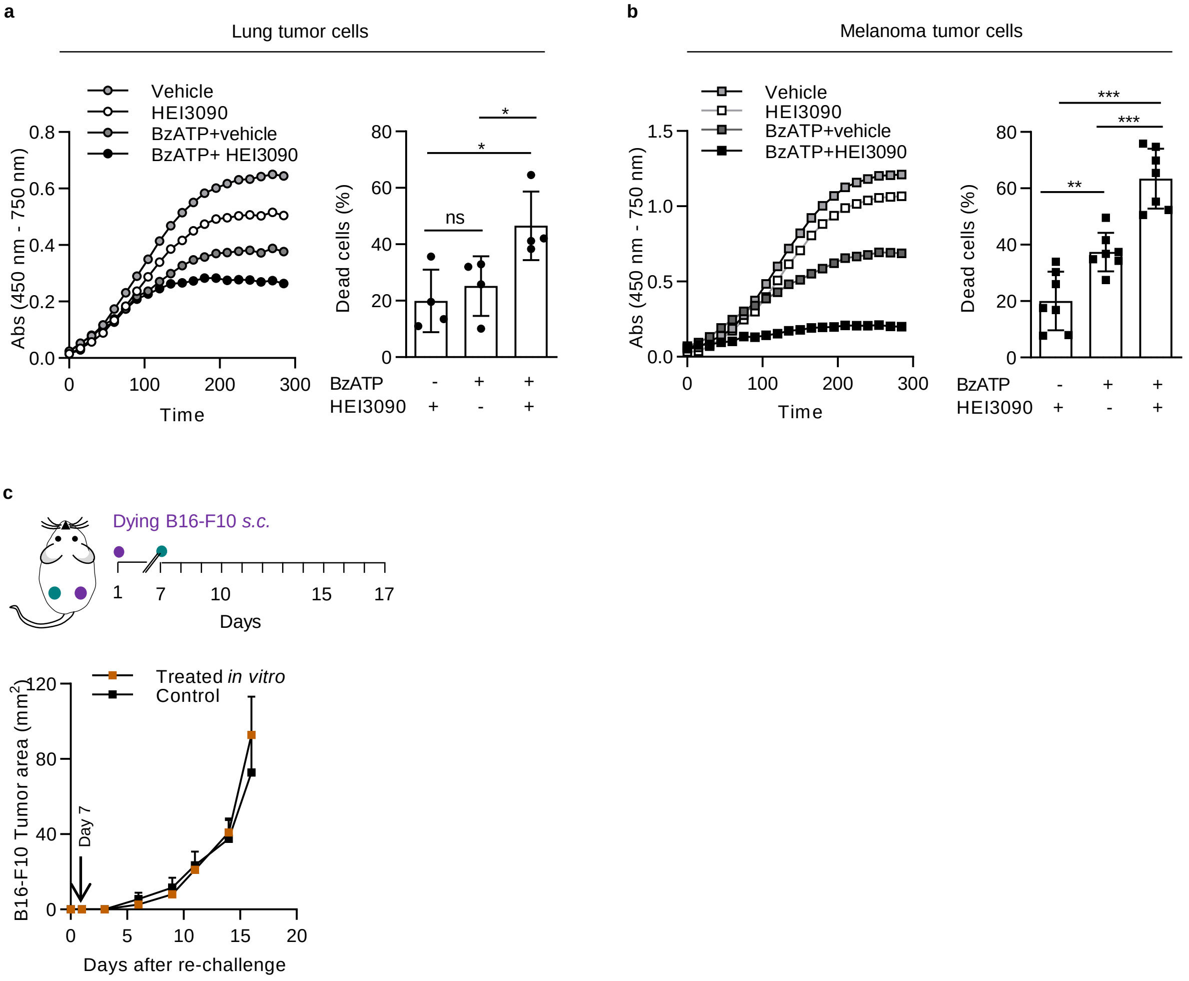

Supplementary Fig. 7: HEI3090 does not promote imunogenic cell death (ICD)

HEI3090-induced LLC (**a**) and B16-F10 (**b**) tumor cell toxicity. Curves showed mean of absorbance (n= 4 independent experiments in **a** and n=7 in **b**, left panel) and graph showed the percentage of dead cells. Data are presented by scatter dot plots showing all points with hatched bar corresponding to the median of dead cells (Two-tailed Mann Whitney test, right panel). **c.** *In vivo* assay for the evaluation of HEI3090 as an inducer of immunogenic cell death. B16-F10 were exposed to 3mM ATP and 50µM HEI3090. WT mice were inoculated s.c. with 1.10^5 dying B16-F10 cells in the right flank (n=6) or PBS as control (n=4). 7 days later, 5.10^5 live B16-F10 cells were injected s.c. in the contralateral and tumor growth in the left flank was measured daily. Curves showed mean tumor area in mm^2 ± SEM (n= 5 mice, Mantel Cox test). The details of the ICD experiment are provided in the methods section. p-values: *p<0.05, **p<0.01 ***p<0.001. Source data are provided as a Source Data file.

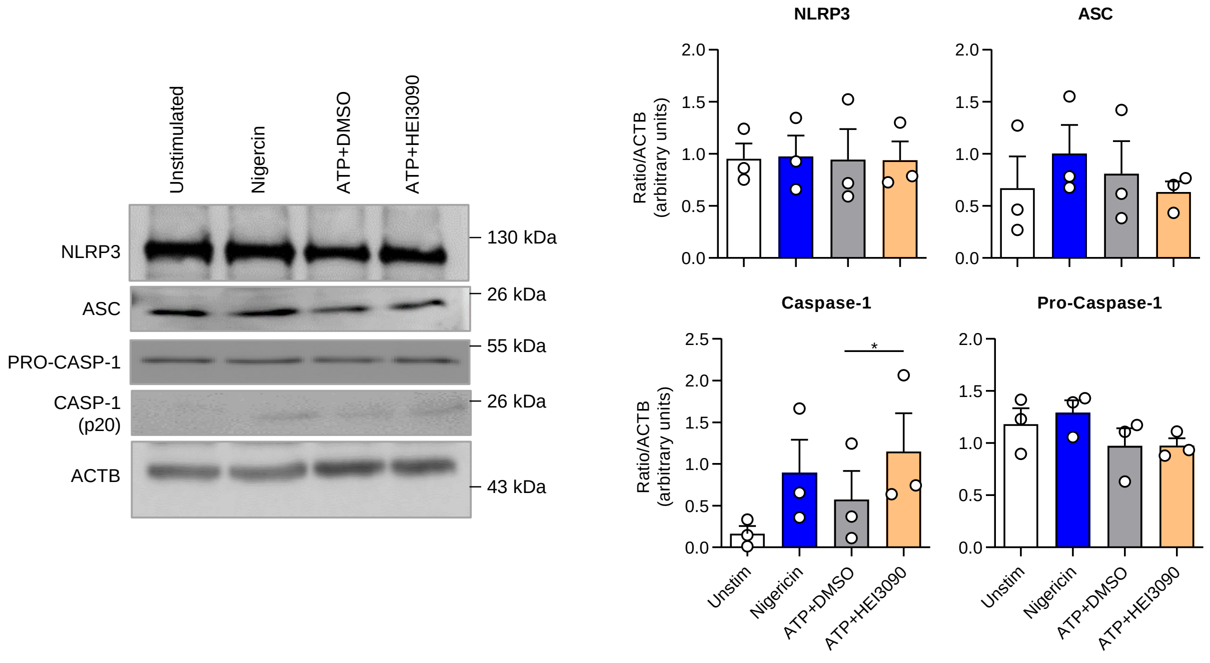

Supplementary Fig. 8: HEI3090 increased eATP-induced caspase-1 cleavage in macrophages
4.10^5 peritoneal macrophages from WT mice were primed for 4h at 37˚C with 100 ng/ml LPS and then stimulated for 30 minutes with 10 µM nigericin or 3 mM ATP with 50 µM HEI3090 or DMSO. **Left panel.** Whole cell protein extracts were analyzed by western blotting by using antibodies recognizing NLRP3, ASC, pro-caspase-1 and active form caspase-1, and β-actin as a loading control. Samples derived from the same experiment and gels/blots were processed in parallel. **Right panel.** Relative band intensities of each protein were assessed to that of β-actin. Data are presented by scatter dot plots, mean ± SEM (n=3, Two-tailed paired t-test).). p-values: *<0.05. Source data are provided as a Source Data file.

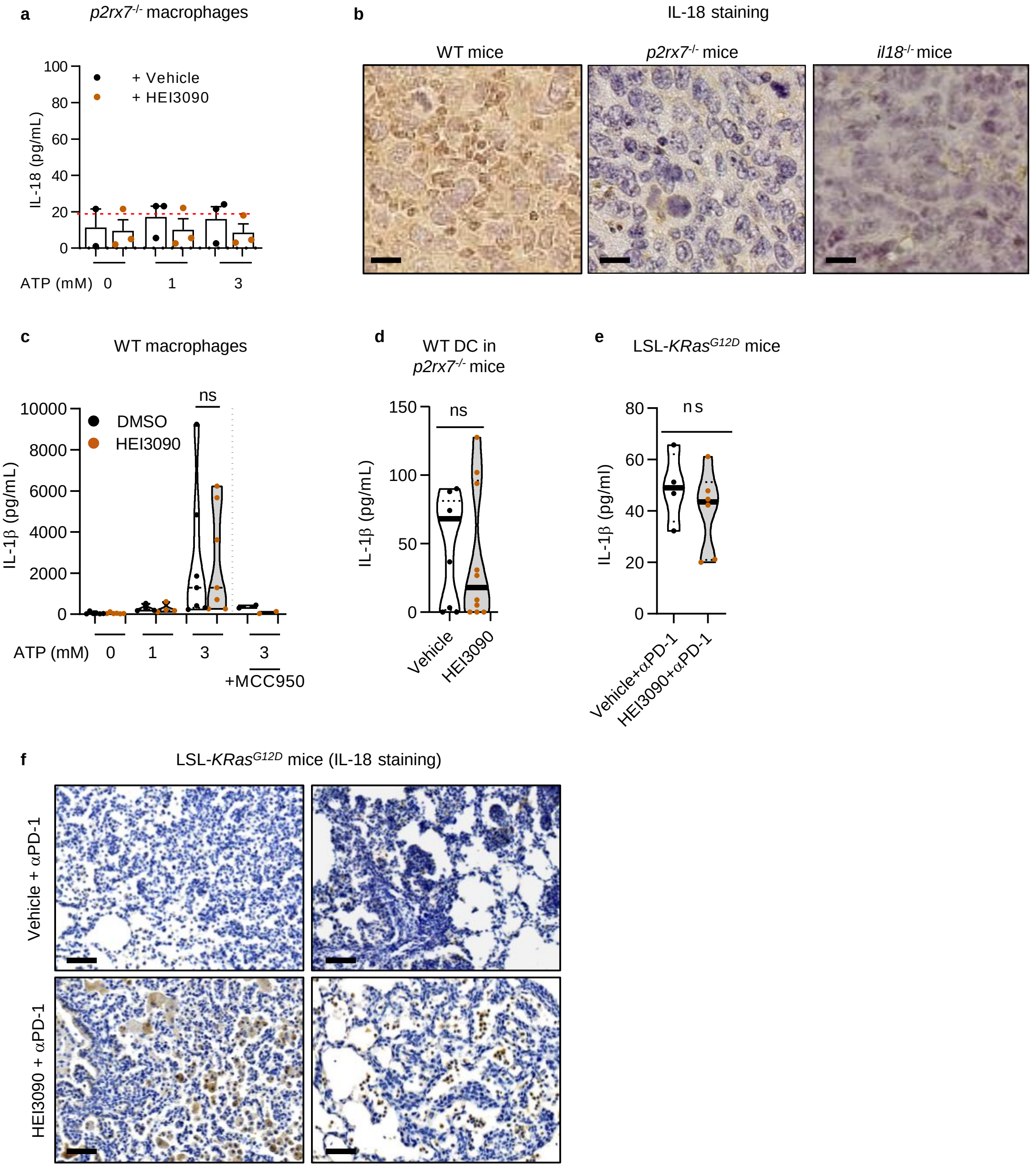

Supplementary Fig. 9: HEI3090-induced IL-18 production required P2RX7 expression

a. Production of IL-18 (ELISA) by peritoneal macrophages isolated from *p2rx7*$^{-/-}$ mice, demonstrating that without P2RX7 expression, HEI3090 is unable to increase IL-18 levels. Data are presented by scatter dot plots (n= 3 mice, Two-tailed Mann Whitney test) **b.** IL-18 staining of LLC tumors collected from WT, *p2rx7*$^{-/-}$ or *il18*$^{-/-}$ mice, demonstrating the specificity of IL-18 antibody. Images are representative of staining of 5 independent mice. Bar= 250nm **c.** Production of IL-1β (ELISA) by peritoneal macrophages isolated from WT mice, demonstrated that HEI3090 was unable to increase IL-1β levels. The specific inhibitor of NLRP3 (MCC950 compound) efficiently inhibits IL-1β production. Vehicle n=6, HEI3090 n=6, ATP 1mM, n= 4, ATP 1mM + HEI3090, n=4, ATP 3mM, n= 7, ATP 3mM + HEI3090, n=7, ATP 3mM + MCC950, n= 2, ATP 3mM + HEI3090 + MCC950, n=2, **d.** Quantification of IL-1β levels from serum of indicated mice confirming that HEI3090 does not modulate the production of IL-1β in *p2rx7*$^{-/-}$ complemented with WT DC. Vehicle n=9, HEI3090 n=10 **e**. Quantification of IL-1β levels from serum of LSL *KRas*^{G12D} mice treated with HEI3090 and αPD-1 antibody. Vehicle + αPD-1, n=4, HEI3090 + αPD-1, n=6. Data are presented by violin plots showing all points with hatched bar corresponding to the median for **c**, **d** and **e** (Two-tailed unpaired t-test) **f.** IL-18 staining from lung of LSL *KRas*^{G12D}, illustrating that IL-18 expression was increased in lung macrophages of HEI3090 + αPD-1 treated mice. Images are representative of staining of 5 independent mice. Bar= 100µm. Source data are provided as a Source Data file.

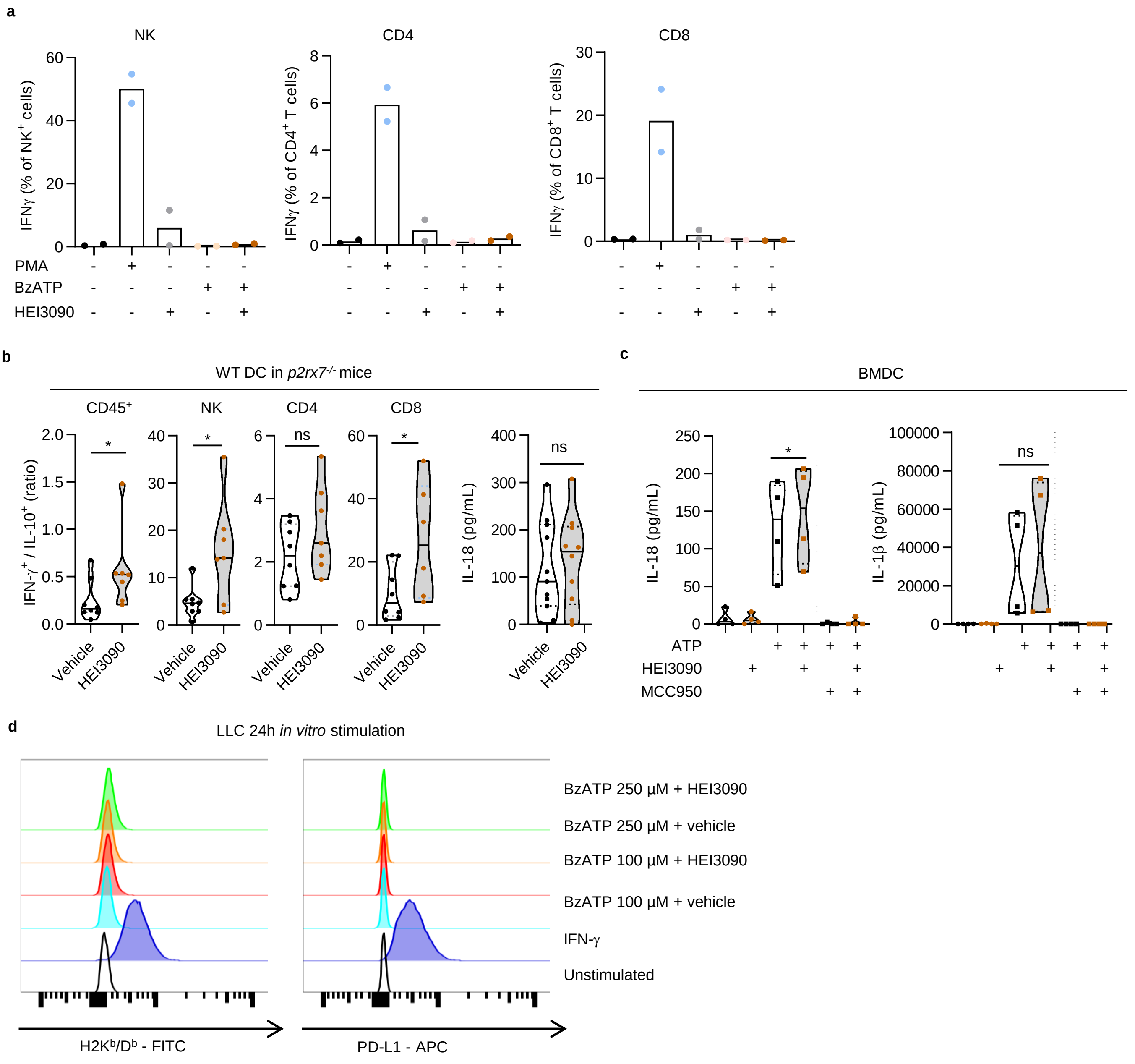

Supplementary Fig. 10: Indirect effect of HEI3090 on IFN-γ production by antitumor immune cells

a. *Ex vivo* IFN-γ production. Splenocytes from WT mice were treated as indicated and IFN-γ production was assayed by flow cytometry in NK, $CD4^+$ and $CD8^+$ T cells. Data are presented by scatter dot plots showing all points. n=2 mice **b.** Purified WT DC were inoculated to *p2rx7*$^{-/-}$ mice i.v. at D-1. At D0, $5x10^5$ LLC cells were injected s.c.. Mice were treated i.p. with vehicle or with HEI3090 daily for 11 days. At D12, mice were sacrificed, sera and tumors were collected for flow cytometry and ELISA analyses, respectively. The ratio of IFN-γ on IL-10 in indicated cells and the concentration of IL-18 in the sera are shown. **c**. BMDC were activated for 4 hours with 100 ng/ml LPS and then stimulated for 30 minutes with 3 mM ATP with or without HEI3090. When indicated, NLRP3 was inhibited for 1 hour with 1 µM of MCC950. IL-18 and IL-1β levels were determined by ELISA. **b** and **c**, data are presented by violin plots showing all points with hatched bar corresponding to the median (**b**. n=10, **c**. n=4, Two-tailed Mann Whitney-test). **d.** MHC-I and PD-L1 expression in LLC tumor cells in response to the indicated treatment. Images are representative of one experiment. p-values: *<0.05. Source data are provided as a Source Data file.

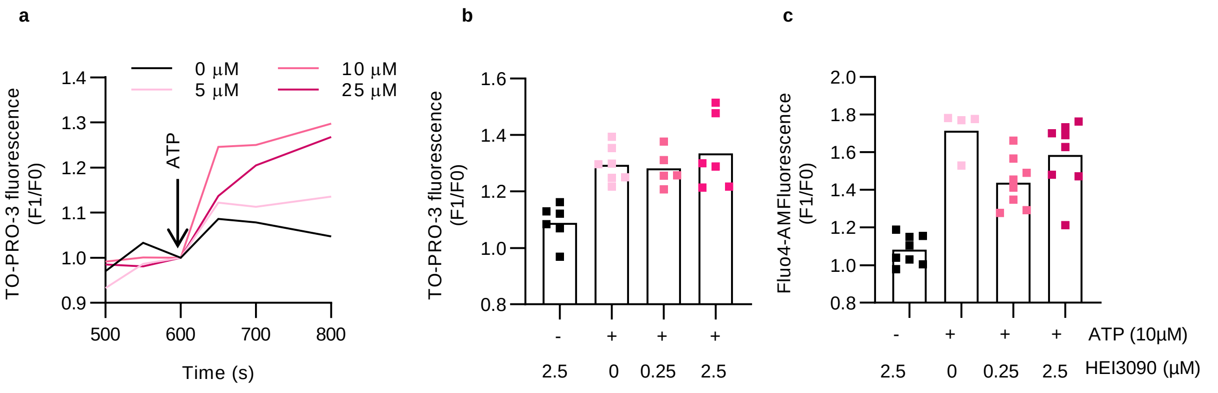

Supplementary Fig. 11: HEI3090 is not a positive modulator for ATP-induced mP2RX4-mediated TOPRO-3 uptake and Ca^{2+} influx in HEK cells

a. HEK293T cells were stably transfected with mouse P2RX7 coding vector and cells were stimulated with various concentrations of ATP. TOPRO-3 uptake over 200 sec was measured. Curve show mean of TO-PRO-3 fluorescence acquired with a spectrophotometer from 2 independent experiments **b.** TOPRO-3 uptake in response to ATP (10 µM) and the indicated doses of HEI3090 150 sec post stimulation. Data are presented by scatter dot plots showing all points from 2 independent experiments with 4 replicates. All points are shown to overcome the high heterogeneity observed between replicates. **c.** Intracellular Ca^{2+} response was measured in Fluo4-AM loaded mP2RX4 HEK cells 50 sec post stimulation. Data are presented by scatter dot plots showing all points. Mean from 2 independent experiments with 4 replicates are shown to overcome the high heterogeneity observed between replicates. Source data are provided as a Source Data file.

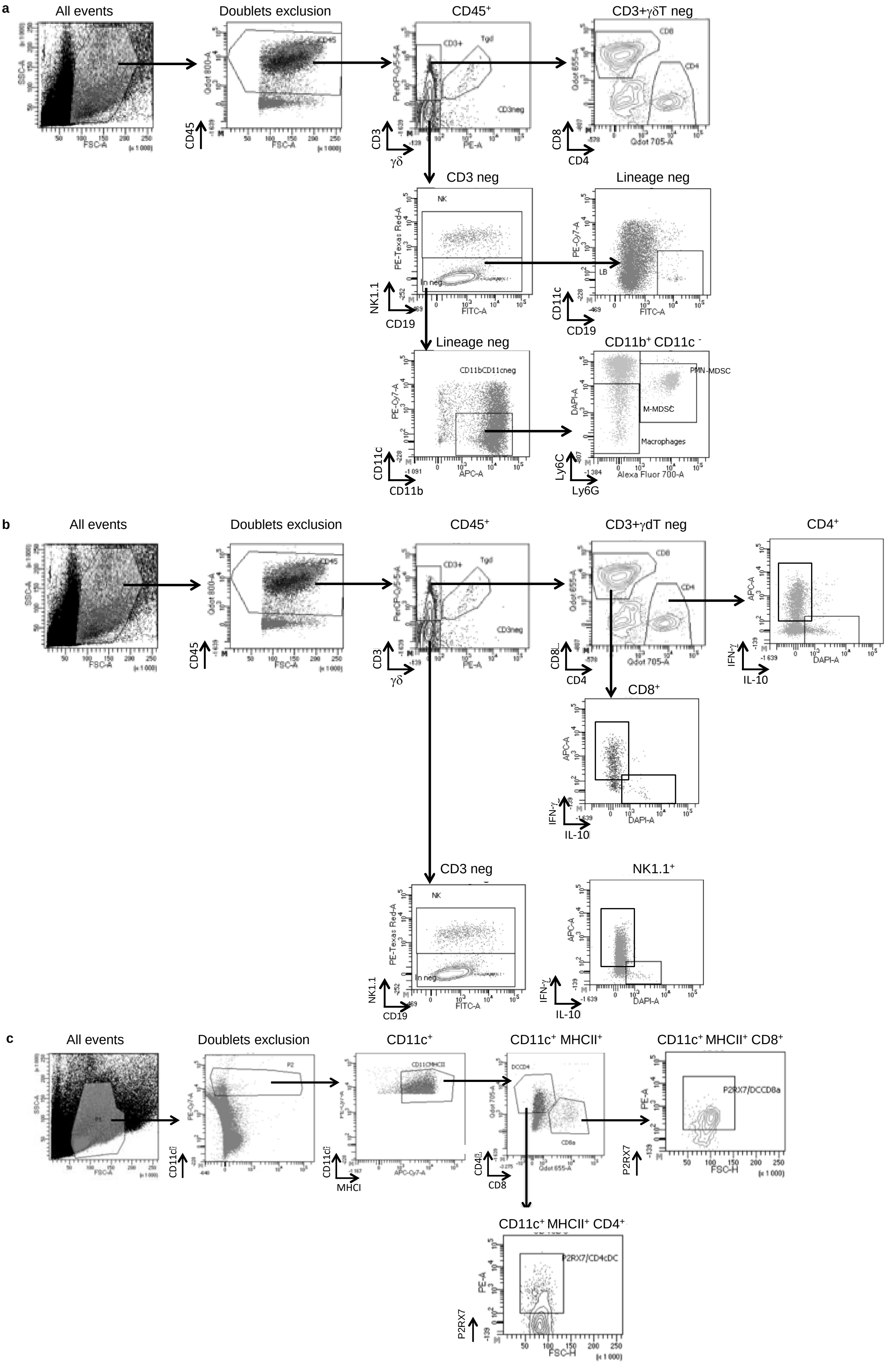

Supplementary Fig.12: Gating strategies

a. Gating strategy for tumor immunophenotyping. **b.** Gating strategy for cytokine production within tumor. **c**. Gating strategy for P2RX7 expression by DC in spleen
